# Lifestyle interventions in primary health care: professional and organizational challenges

**DOI:** 10.1093/eurpub/ckt052

**Published:** 2013-05-30

**Authors:** Therese Kardakis, Lars Weinehall, Lars Jerdén, Monica E. Nyström, Helene Johansson

**Affiliations:** 1 Department of Public Health and Clinical Medicine, Epidemiology and Global Health, Umeå University, SE-901 85 Umeå, Sweden; 2 Department of Learning, Informatics, Management and Ethics, Medical Management Centre, Karolinska Institutet, SE-171 77 Stockholm, Sweden; 3 Center for Clinical Research Dalarna, Primary Health Care Division, SE-791 82 Falun, Sweden

## Abstract

**Background:** Interventions that support patient efforts at lifestyle changes that reduce tobacco use, hazardous use of alcohol, unhealthy eating habits and insufficient physical activity represent important areas of development for health care. Current research shows that it is challenging to reorient health care toward health promotion. The aim of this study was to explore the extent of health care professional work with lifestyle interventions in Swedish primary health care, and to describe professional knowledge, attitudes and perceived organizational support for lifestyle interventions. **Methods:** The study is based on a cross-sectional Web-based survey directed at general practitioners, other physicians, residents, public health nurses and registered nurses (*n* = 315) in primary health care. **Results:** Fifty-nine percent of the participants indicated that lifestyle interventions were a substantial part of their duties. A majority (77%) would like to work more with patient lifestyles. Health professionals generally reported a thorough knowledge of lifestyle intervention methods for disease prevention. Significant differences between professional groups were found with regard to specific knowledge and extent of work with lifestyle interventions. Alcohol was the least addressed lifestyle habit. Management was supportive, but structures to sustain work with lifestyle interventions were scarce, and a need for national guidelines was identified. **Conclusions:** Health professionals reported thorough knowledge and positive attitudes toward lifestyle interventions. When planning for further implementation of lifestyle interventions in primary health care, differences between professional groups in knowledge, extent of work with promotion of healthy lifestyles and lifestyle issues and provision of organizational support such as national guidelines should be considered.

## Introduction

Globally, chronic diseases are the leading causes of death and disability.^1^ A large proportion of these diseases are preventable through reduction of tobacco use, hazardous use of alcohol and unhealthy eating habits and by increasing physical activity.^2^ In Sweden, risky lifestyles are assessed to be responsible for ∼18% of the total burden of disease directly, besides their influence on important factors like high blood pressure and cholesterol.^3^

In the Ottawa Charter,^4^^,^^5^ health care was emphasized to be of particular importance as an arena for health promotion because of its trustworthiness to citizens and range of contact with the overall population. Even though there are examples of successful implementations,^6^^,^^7^ it seems to be difficult to reorient health care toward health promotion.^8^ The fact that only a limited number of consistently executed successful lifestyle interventions in health care are reported,^9^^,^^10^ illustrates the dilemma. Consequently, there is a need for a deeper understanding of how health care can improve its ability to address patient lifestyles, as well as identifying requirements for increased implementation of health orientation within health care.

Changes in complex organizations can be of an emergent character, but are often a result of planned strategic actions.^11^ Moreover, implementation is influenced by the innovation itself, organizational readiness to change, management actions and support, as well as by the adopters’ characteristics.^12^^,^^13^ McLean *et al.*^14^ argues that health reorientation capacities are composed of three dimensions: capacities at the individual, organizational and environmental levels. At the individual level, professional knowledge and commitment can influence the degree to which policies are implemented in daily practice. At the organizational level, primary health care management can control resources, develop structures or foster environments that are either supportive or hostile to implementation efforts. At the environmental level, medical facilities operate within political and economic systems that can have considerable impact on efforts to foster preventive measures.

Thus far, research has to a limited extent focused on the conditions influencing implementation of health orientation in health care. Most of the published studies focus on general practioners,^15–20^ only a few address nurses or other health professionals.^17^^,^^18^^,^^20^^,^^21^ Lack of time,^15–18^^,^^20^^,^^21^ knowledge gaps,^15^^,^^20^^,^^21^ perceived priorities^17^^,^^21^ and doubts about the effectiveness of lifestyle interventions are identified as common obstacles at the individual level.^15^^,^^16^^,^^21^ The dominant organizational obstacles are reported to be ambiguous objectives and protocols and lack of appropriate structures and referral options.^16–21^

Even when health orientation is part of the health care agenda, knowledge about how to support healthier lifestyle, the extent of involvement by health professionals and organizational support for health orientation has not received much attention. This article aims to investigate the extent of health professional work with lifestyle interventions in primary health care in Sweden, and to describe the knowledge, attitudes and organizational support that are available for lifestyle interventions. The study will focus on habits related to tobacco use, hazardous use of alcohol, unhealthy eating habits and insufficient physical activity.

The present study is a part of a larger research project ‘National Guidelines for health promotion in health care – the challenge to apply scientific evidence in clinical practice’, which has the aim to study how new knowledge can be implemented and sustained in health care organizations. This larger project investigates the development and implementation of the National Guidelines for Lifestyle Interventions in Sweden. The implementation is studied at different levels of primary health care in two regions. The research project started in year 2010, and will end in 2013.

## Methods

This cross-sectional descriptive study was carried out in 2011 among nurses and physicians in primary health care. Data were collected through a Web-based questionnaire.

### Setting

The Swedish health care system is organized at national, regional and local levels. At the national level, the Ministry of Health and Social Affairs establishes principles and guidelines for care and sets the primary agenda for health care. At the regional level, the responsibility for financing and provision of health care is decentralized to 21 health care regions (county councils). The directly elected county councils have full budgetary responsibility for providing health care to all citizens. By Swedish law, health promotion and prevention is a health care responsibility, regardless of level of care. Most of the efforts are, however, undertaken in primary care. At the local level, municipalities are responsible for social services. The Swedish health care system is primarily funded by taxes. In addition to tax revenues, financing of health care services is supplemented by governmental grants and user fees.^22^

### Sample and survey

Two health care regions were purposively selected for accessibility and comparability. Both of them are large geographically, situated in the north of Sweden and sparsely populated. Moreover, the two regions have similar sociocultural context and health care systems. After consent of the chief executive officer/public health director, the Human Resources offices were contacted to obtain data on potential participants, including names, e-mail addresses and worksites. A Web-based questionnaire was distributed as a link in an ‘invitation to participate’ e-mail, directed to all permanently employed professionals in community medicine within 65 primary health care centres, i.e. registered nurses, public health nurses, physicians, general practitioners and residents. Five reminders were sent over a 3-month period. A Web-based questionnaire was developed based on a literature review. The questionnaire was pilot-tested on a sample of eight persons to assess its face validity. Because the study is part of a larger research project, only a portion of the questionnaire (16 items of 49) was used. Of the 16 items, four measured attitudes, two measured knowledge of lifestyle intervention methods (of which one had four sub-items), three measured the extent to which professionals and primary health care centres work with patient lifestyles (of which one had four sub-items) and seven measured perceived organizational support. A 5-point Likert scale was used to quantify the responses (item responses ranged from ‘completely disagree’ to ‘completely agree’).

### Data analyses

SPSS software was used for data analysis. The two Likert scale responses ‘completely agree’ and ‘agree to a great extent’ were clustered into one category and considered to support the questionnaire items. The remaining responses were considered to support the items to a lesser extent or not at all: ‘partly agree’, ‘partly disagree’ and ‘completely disagree’. Analyses of differences between professional groups, work experience (0–10 years vs. >11) and gender were conducted. Gender differences were analyzed only among physicians because the nursing groups consisted of 96% women. Only one statistical significant difference was found (for gender) and this finding is not shown in the tables. Statistical significance was determined by Pearson chi-square tests. *P*-values <0.05 were considered significant.

### Ethical considerations

Ethical approval was obtained from the Regional Ethical Committee in Umeå (Dnr. 2011-64-31M). All respondents were informed of the purpose of the research, the person to contact for further information and that confidentiality would be secured throughout the research process.

## Results

### Characteristics of respondents

The response rate was 49% (315 participants) from 61 primary health care centres. Fifty-two percent of the nurses (*n* = 215) and 42% of the physicians (*n* = 100) responded. Of the 315 participants, 81% were women. The largest professional group was nurses (68%) and included registered nurses and public health nurses. The physician group (32%) included general practitioners, other qualified physicians and residents. Sixty percent of the participants had >10 years of work experience.

### Attitudes

Seventy-nine percent of all participants considered it important that health professionals work with patient lifestyles (table 1). Eighty-nine percent considered work with patient lifestyles to be compatible with the overall aims and objectives of primary health care. However, the numbers varied between 74% (registered nurses) and 94% (general practitioners) (*P* = 0.004). As shown in table 1, 50% of participants stated that as a group they work extensively with promotion of healthy lifestyles in their health care centre. However, there were large differences among the professional groups (*P* = 0.022). Registered nurses were most likely (66%), and residents least likely (25%), to report extensive promotion of healthy lifestyles at their health care centre. Health professionals with longer work experience reported more extensive promotion of healthy lifestyles (55%) compared with those with shorter work experience (43%) (*P* = 0.045). Eighty-five percent of all professionals expressed a need to develop health care centre work with promotion of healthy lifestyles (table 1). Public health nurses (89%) expressed this need more often than did physicians (70%) (*P* = 0.034).

**Table 1:** Attitudes toward promotion of healthy lifestyles, % of health care professional who agree to a high extent

Statements	At my PHCC,^a^ we consider it important to promote healthy lifestyles for our patients		At my PHCC, we work extensively with the promotion of healthy lifestyles among our patients		I perceive tasks like promotion of healthy lifestyles as compatible with overall PHC^b^ aims and objectives		According to me, there is a need to develop work with health promotion regarding PHC	
Characteristics	%	*P*-value	%	*P*-value	%	*P*-value	%	*P*-value
Profession		0.973		**0.022**		**0.004**		**0.034**
Registered nurse	77		66		74		86	
Public health nurse	80		49		92		89	
Physician	78		55		90		70	
Resident^c^	75		25		75		82	
General practitioner	78		56		94		81	
Years in profession		0.908		**0.045**		0.535		0.910
0–10	78		43		87		85	
≥11	79		55		90		85	
Total	79		50		89		85	

a: PHCC: primary health care centre.

b: PHC: primary health care.

c: Resident in community medicine.

Values in bold indicate statistical significance of differences because they are *P* < 0.05.

### Knowledge of lifestyle intervention methods for disease prevention

Of all participants, 76% believed that as a group they possess a thorough knowledge about lifestyle intervention methods for disease prevention (table 2). Public health nurses were most confident about collective knowledge at the health care centre (83%), whereas residents were least confident (57%) (*P* = 0.008). Health professionals considered that the area where they themselves had the greatest knowledge was methods to counteract insufficient physical activity (84%). The least knowledge was reported for hazardous use of alcohol (66%). Differences were seen between the professional groups, particularly regarding unhealthy eating habits (*P* ≤ 0.001). Knowledge of intervention methods regarding unhealthy eating habits also differed by work experience (*P* ≤ 0.001). Significant differences by experience (*P* = 0.048) and gender (*P* = 0.028, data not showed) were seen for hazardous use of alcohol. General practitioners reported the greatest knowledge of lifestyle intervention methods, and registered nurses reported the lowest.

**Table 2:** Knowledge of lifestyle intervention methods for disease prevention, % of health care professionals who agree to a high extent

Statements	At my PHCC,^a^ we have a thorough knowledge of how we can promote healthy lifestyle habits among our patients		I have a thorough knowledge of disease prevention methods concerning tobacco use		I have a thorough knowledge of disease prevention methods concerning hazardous use of alcohol		I have a thorough knowledge of disease prevention methods concerning unhealthy eating habits		I have a thorough knowledge of disease prevention methods concerning insufficient physical activity	
Characteristics	%	*P*-value	%	*P*-value	%	*P*-value	%	*P*-value	%	*P*-value
Profession		**0.008**		0.346		0.840		<**0.001**		0.071
Registered nurse	69		63		60		71		71	
Public health nurse	83		72		66		88		87	
Physician	68		76		68		70		80	
Resident^b^	57		75		61		46		75	
General practitioner	72		84		72		75		91	
Years in profession		0.050		0.083		**0.048**		<**0.001**		0.137
0–10	70		68		59		69		80	
≥11	80		77		70		85		86	
Total	76		73		66		79		84	

a: PHCC: primary health care centre.

b: Resident in community medicine.

Values in bold indicate statistical significance of differences because they are *P* < 0.05.

### Extent of work with lifestyle interventions

At the individual level, 59% of respondents reported that promotion of healthy lifestyles was a substantial part in their own work with patients (table 3). General practitioners (66%) and public health nurses (64%) indicated that promotion of healthier lifestyle was a more substantial part of their work compared with other professional groups (*P* = 0.081). Residents reported the lowest level (43%) of promotion activities. Health professionals with longer work experience (66%) reported more work with patients’ lifestyle than health professionals with shorter experience did (49%) (*P* = 0.002). Seventy-seven percent of all professionals would like to work more to support promotion of healthy lifestyles among their patients. Public health nurses were most favourable toward this (83%) (*P* = 0.040).

**Table 3:** Extent of work with promotion of healthy lifestyle habits, % of health care professionals who agree to a high extent

Statements	The promotion of healthy lifestyle habits among my patients is a substantial part of my duties		I work to a great extent with promotion of healthy lifestyle habits concerning tobacco use		I work to a great extent with promotion of healthy lifestyle habits concerning the hazardous use of alcohol		I work to a great extent with promotion of healthy lifestyle habits concerning unhealthy eating habits		I work to a great extent with promotion of healthy lifestyle habits concerning insufficient physical inactivity		I would like to work to a greater extent with promotion of healthy lifestyles among my patients	
Characteristics	%	*P*-value	%	*P*-value	%	*P*-value	%	*P*-value	%	*P*-value	%	*P*-value
Profession		0.081		**0.010**		0.515		**0.015**		**0.016**		**0.040**
Registered nurse	54		46		43		51		57		69	
Public health nurse	64		63		58		72		75		83	
Physician	48		70		55		60		48		68	
Resident^a^	43		79		57		46		79		68	
General practitioners	66		91		63		72		94		69	
Years in profession		**0.002**		0.148		0.093		0.112		0.481		0.279
0–10	49		62		50		61		73		80	
≥11	66		70		60		70		76		75	
Total	59		66		56		66		75		77	

a: Resident in community medicine.

Values in bold indicate statistical significance of differences because they are *P* < 0.05.

Seventy-five percent of all professionals assessed insufficient physical activity to be the most common lifestyle habit to be addressed in their work. The hazardous use of alcohol was reported to be given least attention (56%). The extent of how much effort was devoted to the four lifestyle habits differed significantly between the professional groups (tobacco use *P* = 0.010; unhealthy eating habits *P* = 0.015; insufficient physical inactivity *P* = 0.016), except for the hazardous use of alcohol (*P* = 0.515). General practitioners indicated the greatest extent of work with promotion of healthy lifestyles, whereas registered nurses indicated the lowest.

### Organizational support structures

Among all health professionals, 71% experienced management at the health care centre as positive toward promotion of healthy lifestyles (table 4). Twenty-two percent stated that professionals within the health care centre collaborate with stakeholders, such as municipalities and associations, to improve patient lifestyles.

**Table 4:** Organizational support structures for clinical work with patient lifestyles, % of health professionals who agree to a high extent

Statements	At my PHCC,^a^ management is positive toward our work with promotion of healthy lifestyles		At my PHCC, we collaborate with other stakeholders such as municipalities and community associations about our patients’ lifestyles		At my PHCC, there are incentives to promote healthy lifestyles among our patients		At my PHCC, there are local guidelines/care programs on how we should promote healthy lifestyles among our patients		At my PHCC, there are many factors that facilitate the promotion of healthy lifestyles among our patients		At my PHCC, there are many factors that hinder the promotion of healthy lifestyles among our patients		I perceive there is a need for National CPGs^b^ for lifestyle interventions in the work at my PHCC	
Characteristics	%	*P*-value	%	*P*-value	%	*P*-value	%	*P*-value	%	*P*-value	%	*P*-value	%	*P*-value
Profession		0.782		0.207		0.465		0.618		0.154		0.729		**0.049**
Registered nurse	80		37		60		63		37		17		77	
Public health nurse	71		22		57		51		42		17		84	
Physician	68		20		63		50		43		12		65	
Resident^c^	68		14		50		50		14		18		68	
General practitioner	72		19		72		72		38		25		75	
Years in profession		0.489		0.829		0.111		0.948		0.075		0.861		0.783
0–10	74		22		54		51		27		18		79	
≥11	70		23		63		52		37		17		78	
Total	71		22		59		51		33		17		78	

a: PHCC: primary health care centre.

b: CPG: clinical practice guidelines.

c: Resident in community medicine.

Values in bold indicate statistical significance of differences because they are *P* < 0.05.

Within the health care centres, 59% reported incentives for working with patient lifestyles. Thirty-three percent of all professionals indicated that many factors facilitate work with patient lifestyles at their health care centres. However, the figures varied between professional group (*P* = 0.154) and by work experience (*P* = 0.075). Seventeen percent of the professionals stated that there are many obstacles to this kind of work. The existence of local guidelines to guide work with the promotion of healthy lifestyles was reported by 51% of all professionals. Seventy-eight percent perceived a need for national guidelines regarding methods for lifestyle interventions in clinical practice; however, this figure varied between professional groups (*P* = 0.049).

## Discussion

It is a common experience that reorientation of health care toward a more pronounced health focus is a challenging task. Obviously, relatively few lifestyle interventions have been consistently implemented in health care.^9^^,^^10^ In this study, we explored the extent of health professionals’ work with lifestyle interventions, and their attitudes, knowledge and organizational support for this work.

The health professionals’ attitudes are of key importance for successful implementation of lifestyle interventions.^12–14^ We found that, in general, health professionals have positive attitudes toward lifestyle interventions in health care. A majority considered promotion of healthy lifestyles to be in line with the overall aims of primary health care, and an important mission at the primary health care centre. These results are consistent with previous findings.^15^^,^^17^^,^^19^^,^^21^

The health professionals’ knowledge is also an important prerequisite for implementation and an indication of the capacity for health care reorientation.^13^^,^^14^ Previous studies noted that general practitioners’ lack of knowledge in nutrition and nurses’ lack of knowledge in obesity management methods can be obstacles to lifestyle interventions.^15–19^^,^^21^ Most respondents in our study reported a thorough knowledge of methods addressing tobacco use, hazardous use of alcohol, unhealthy eating habits and insufficient physical activity at an individual level and as members of their health care centre group. This general level of knowledge indicates a potential to sustain implementation of health orientation in Swedish primary health care. However, the level of knowledge differed between professional groups and by duration of work experience. This variation was particularly large concerning unhealthy eating habits and emphasizes the importance of mapping specific needs to identify knowledge gaps. Another important finding was the significant difference in knowledge of intervention methods for prevention of hazardous use of alcohol between male and female physicians. However, this result has not previously been described and needs to be further examined.

This study aimed to review the extent of professional work with lifestyle interventions in primary health care. Half of the health professionals believed that they, as a group, already worked extensively with lifestyle interventions at their health care centre. Fifty-nine percent of the participants declared that lifestyle interventions constituted a substantial part in their individual work with patients. Despite these numbers, a majority would like to do more work with patient lifestyles and perceived a need to develop health care centre efforts to improve the promotion of healthy lifestyles. This finding is consistent with previous research.^18^ Greenhalgh *et al.*^13^ suggest that a potential innovation is more likely to be adopted when staff perceive the current situation as un-satisfying. Health professional willingness to be more involved and develop preventive work in everyday practice might therefore be an indicator of readiness for change both at the individual and group level.

Another important finding was the differences between professional groups concerning the extent of work with lifestyle interventions. Registered nurses reported a low involvement in lifestyle intervention work, and only one-fourth of the residents believed that professionals at the health care centres worked extensively with patients’ lifestyles (table 1). An explanation for this might be a lower level of knowledge about lifestyle intervention methods among both nurses and residents. It should be noted that hazardous use of alcohol was the least addressed lifestyle habit, both in terms of extent of work and in terms of knowledge. A possible explanation might be difficulties to address hazardous alcohol use at the same time when patients’ other lifestyle issues are discussed. Geirsson *et al.*^23^ found that both general practitioners and nurses rate their counselling skills for reducing alcohol consumption lower than for counselling in other areas, such as smoking. Likewise, Ampt *et al.*^15^ show that general practitioners differ in their attitudes toward assessing alcohol consumption, and report that some believed screening was possible only within a specific health check. The lack of effort here noted is in sharp contrast to the recently reported need for active efforts, where lifestyle interventions for reduced alcohol consumption are assessed to be the most important to develop.^24^ Obviously, lifestyle interventions to reduce hazardous use of alcohol need further consideration.

Health professionals reported organizational support for lifestyle interventions in terms of management and incentives. Only 17% experienced factors at the health care centre to hinder their lifestyle intervention work. An important challenge to improve patient lifestyle would be interventions where primary health care centres collaborate with other stakeholders. Johnson and Paton^25^ point out that in the broadest sense, health orientation in general practice should involve the community being served. Previous research describes lifestyle promotion to be negatively influenced if primary health care centres are isolated from other community services and lack knowledge of external referral options.^20^^,^^21^ In the current study, 22% of health professionals reported external collaboration, suggesting that additional relations might enhance lifestyle interventions.

Seventy-eight percent of the respondents expressed a need for national guidelines for lifestyle interventions, even though 50% already had such local guidelines. Johansson *et al.*^18^ identify lack of guidelines as a barrier for health promotion. Similarly, Ribera *et al.*^20^ found that lack of a structured approach and common criteria are obstacles to health promotion. As mentioned previously, McLean *et al.*^14^ emphasizes how management can develop supportive structures for implementation. The present findings indicate the need for better guidance to support lifestyle interventions. An important contribution will be the National Guidelines for Lifestyle Interventions^26^ developed by the Swedish National Board of Health and Welfare and published a few months after data collection for this study. These guidelines are now being implemented.

### Methodological considerations

The number of items in the questionnaire was limited to obtain a satisfying response rate. However, we believe that the items captured enough relevant information to answer the research questions posed. Studies of this nature in general have a low response rate, thus interpretations must be made with caution. The overall response rate in the present study was 49% and differed between nurses (52%) and physicians (42%). Cook *et al.*^27^ found a declining response rate for questionnaires to health care professionals, with a mean response rate of 56%, and no significant differences between types of surveys. Individuals interested in health orientation may answer at a greater rate than others. Some individuals report that they do not respond because of lack of time and the large number of questionnaires sent to them, but to the best of our knowledge, a number of non-responses also occurred because employee data are infrequently updated by the health care providers (e.g. long-term sick leave, leave of absence and cessation of employment). If data were up-to-date, such individuals would have been excluded from the sample group and would not have received the survey. Each of these factors might potentially have influenced the results and have some impact of the study generalizability.

## Conclusions

The main finding of our study is that the health professionals to a large extent have a positive attitude and thorough overall knowledge about lifestyle intervention methods. Furthermore, they perceive support from primary health care managers for work with promotion of healthy lifestyles. These findings may be interpreted as indications of readiness in primary health care to support implementation of health-oriented interventions. However, the fact that professional groups differ in knowledge and actual involvement needs to be considered when planning for changes. Using surveys to identify points of departure for different professional groups will aid in the planning of educational efforts, as well as identify needed support such as national guidelines. An extended collaboration with other stakeholders around patient lifestyles also seems to be an important step to increase further development of lifestyle interventions in primary health care.
